# Supplementary material for: Adult Diel Locomotor Behaviour in the Agricultural Pest Plutella xylostella Reflects Temperature-Driven and Light-Repressed Regulation Rather than Coupling to Circadian Clock Gene Rhythms
Source: Insects. 2025 Feb 8;16(2):182. doi: 10.3390/insects16020182 (PMC11856205; doi:10.3390/insects16020182)
Supplement: Supplementary file 1 [file insects-16-00182-s001.zip › insects-3429499-supplementary.pdf]

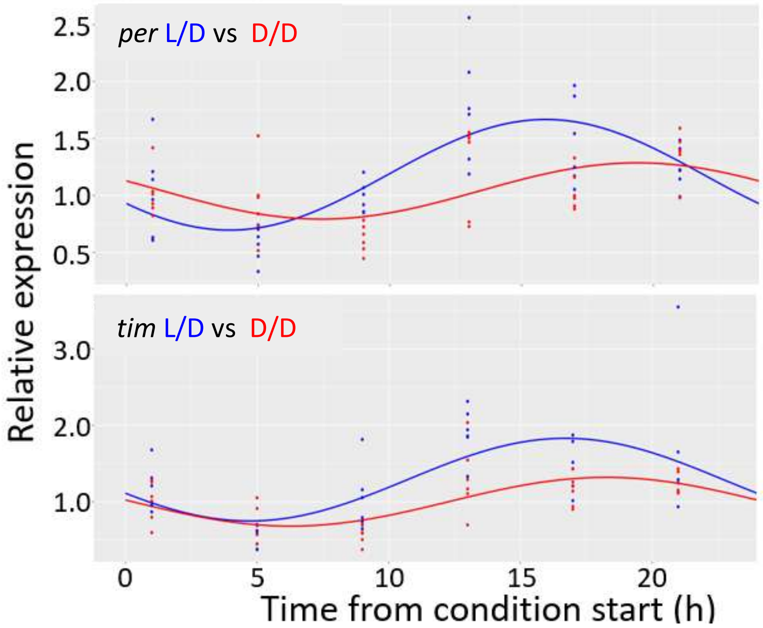

Rhythmic feature	L/D vs D/D	*P* value
Rhythmicity	1.54^{-6} vs 1.35^{-3}	n/a
Mesor	1.18 vs 1.04	0.070
Amplitude*	0.49 vs 0.25	0.032
Phase Peak*	15.90 vs 19.40	0.011

Rhythmic feature	L/D vs D/D	*P* value
Rhythmicity	1.25^{-4} vs 4.44^{-5}	n/a
Mesor*	1.29 vs 1.00	0.005
Amplitude	0.54 vs 0.32	0.122
Phase Peak	16.72 vs 18.27	0.269

Supplemental Figure S1. Comparative analysis of *per* and *tim* L/D and D/D transcript rhythms by cosine fitting. Circa Compare cosinusoidal curve fitting against individual data points of *per* and *tim* data from Figure 1 compared between L/D and D/D conditions. *P* values for comparisons between rhythmic features of curve shown in respective tables to right of plots. Relative expression levels calculated from qPCR data using the 2–ΔΔCt method and * against feature name indicates significant differences between L/D and D/D conditions. Rhythmicity *P* value shown in middle column, equates to the *P* value of data's fit to cosinudsoidal curve, Mesor equates to a rhythm-adjusted mean, Amplitude is a measure of amount of change between peak and trough of calculated curve. Time points refer to L/D zeitgeber time (ZT) and D/D circadian time (CT) time points with phase peak equating to such respectively.

ANOVA	*per* L/D	*per* D/D	*tim* L/D	*tim* D/D
P value	*P* <0.0001	*P* <0.001	*P* <0.001	*P* <0.0001

Time points	Sig	Sig	Sig	Sig
ZT1/CT1	ab	ab	ab	ab
ZT5/CT5	b	b	ab	ac
ZT9/CT9	bc	bc	b	a
ZT13/CT13	d	a	a	b
ZT17/CT17	acd	ac	ab	bc
ZT21/CT21	acd	ac	a	b
2-way ANOVA	*per* L/D vs D/D	*P* value	*tim* L/D vs D/D	*P* value
Condition	2.848	*P* <0.01	7.330	*P* <0.01
Time	42.38	*P* <0.0001	41.86	*P* <0.0001
Interaction	14.18	*P* <0.01	5.789	*P* >0.05

Supplemental Table S1. Comparative analysis of *per* and *tim* L/D and D/D transcript rhythms by ANOVA. One and two-way ANOVA of adult *P. xylostella per* and *tim* expression against time and L/D and D/D conditions. Top: ANOVA of the impact of time on relative *per* or *tim* expression under either L/D or D/D conditions with Tukey's post-hoc tests comparing time points. Bottom: 2-way ANOVA of the effects of time and condition (L/D vs D/D) on relative *per* or *tim* expression.

A.

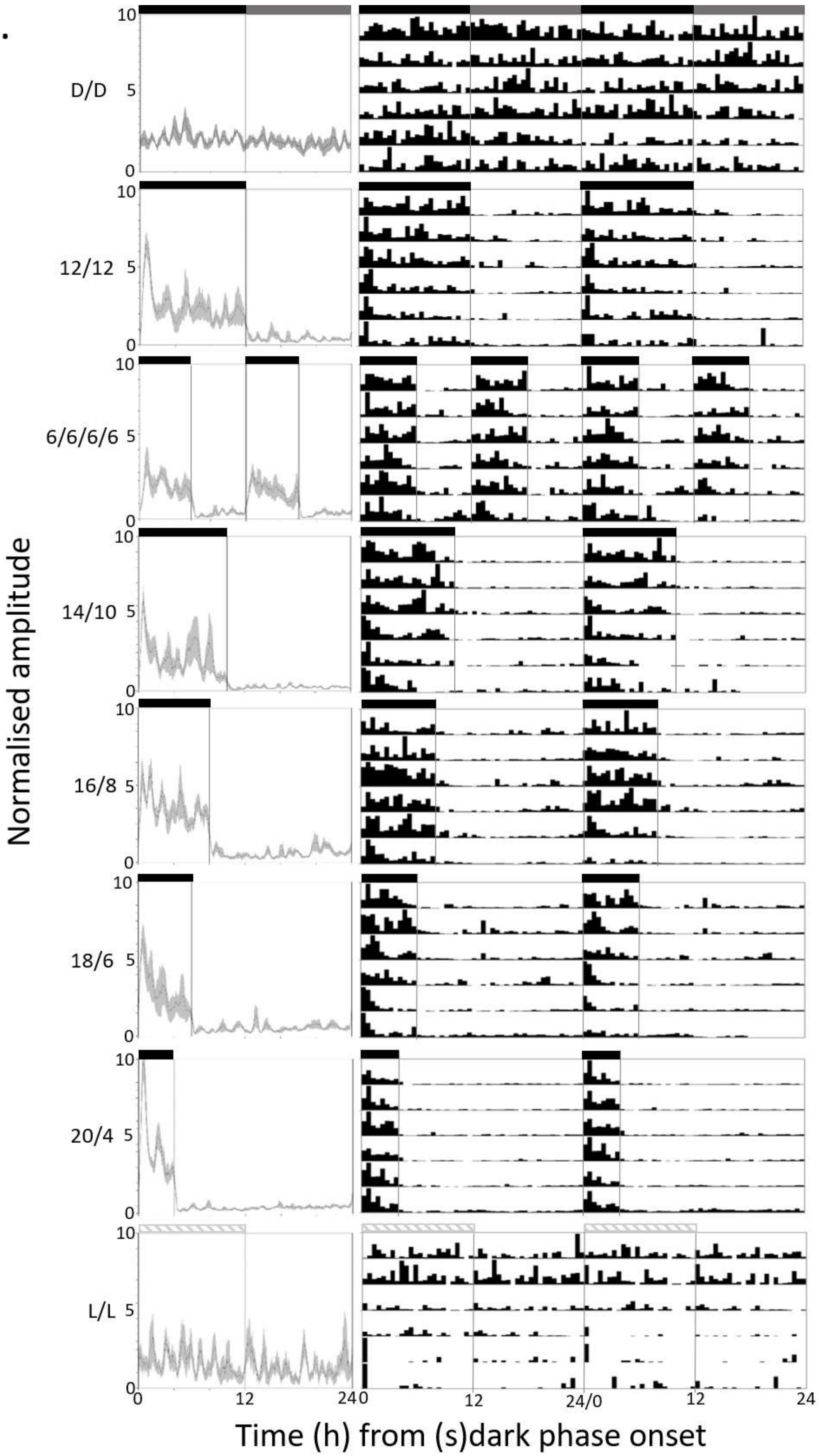

D/D
12/12
6/6/6/6
14/10
16/8
18/6
20/4
L/L
Normalised amplitude
0
5
10
0
12
24
12
24/0
24
Time (h) from (s)dark phase onset

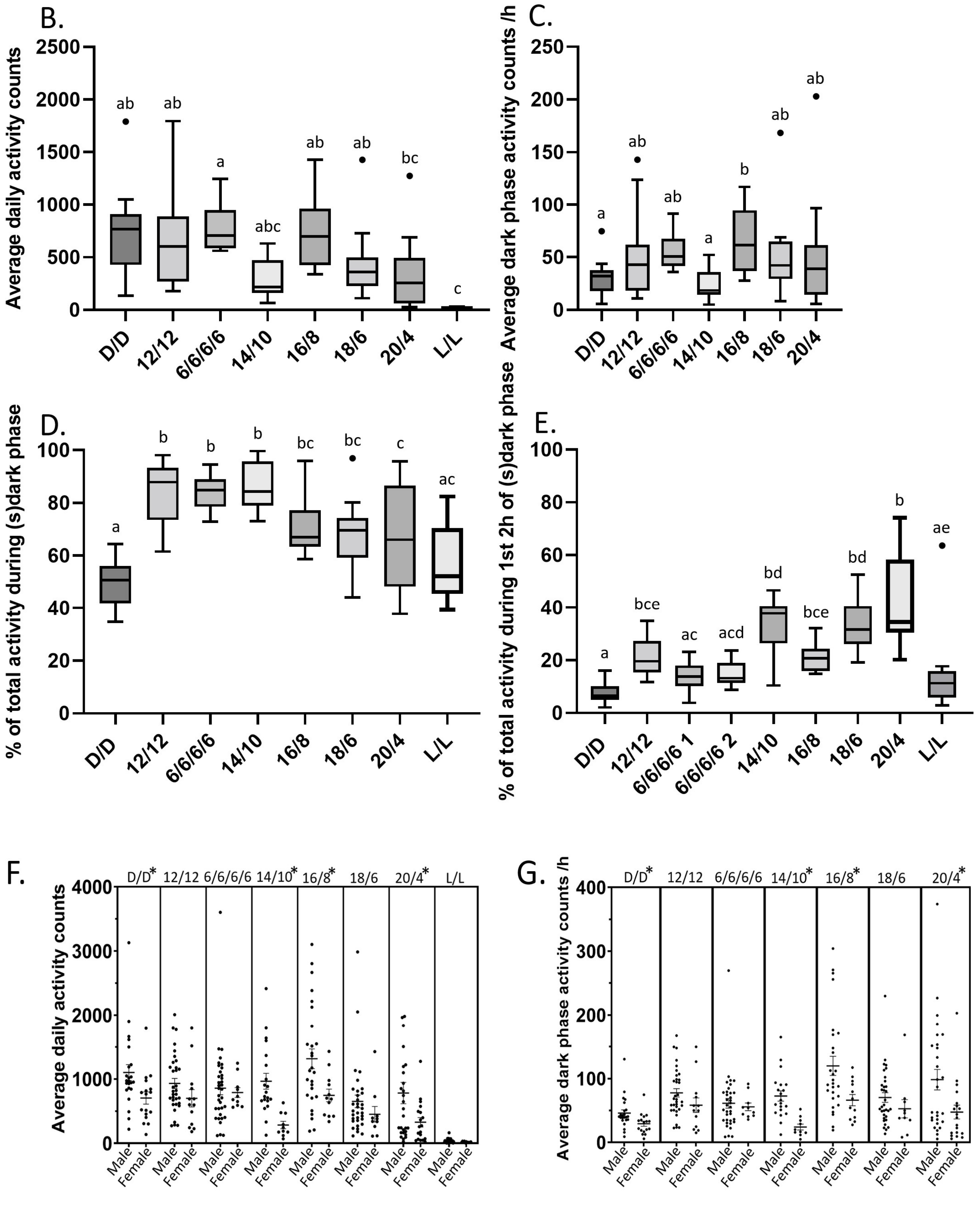

Supplemental Figure S2. Locomotor behavioural analysis of adult female P. xylostella under different lighting conditions.A) Left: Normalized average ± SEM daily activity profiles over 6 day intervals for *P. xylostella* adult females. Time is plotted along x-axis in hours (h), starting at the onset of dark or subjective dark phase with normalized activity

indicated by the y-axis. Right: double-plotted actograms of normalized average activity of moths over 6 days in 30 minute bins. The horizontal bars along the top of individual activity profiles and actograms indicate dark phase (black), subjective dark phase (grey cross hatching), light phase (white) and subjective light phase (grey). B) total average daily activities, C) average hourly dark phase activity, D) average % of total activity occurring during (s)dark phase, E) average % of total activity occurring during first 2 h of (s)dark phase. Data points outside Tukey range (1.5XIQR (Interquartile range)) are shown. Kruskal-Wallis and Dunn's multiple comparison post hoc test results show significant differences from each respective data set through letter grouping system. F) & G) Female *P. xylostella* produce significantly less activity counts than males in multiple light cycle conditions. Nested individual value scatter plots for each light cycle condition between male and females with horizontal bar showing mean with SEM error bars. F) shows total average activity over 24h of adult P. xylostella, G) shows total dark phase average activity divided by dark phase period in h. * shows when male and female activity is significantly different

Female Condition (n)	% rhythmic	Period length (h)	RRP
D/D (17)	17.6	25.25 ±0.7	1.05 ±.01
12/12 (13)	76.9	24.15 ±0.4	1.41 ±.16
6/6/6/6 (10)	80.0	23.9 ±0.2	1.40 ±.07
14/10 (10)	90.0	23.8 ±0.1	1.38 ±.06
16/8 (12)	91.7	25.1 ±0.9	1.16 ±.04
18/6 (10)	40.0	23.6 ±0.1	1.24 ±.08
20/4 (19)	89.5	24 ±0.0	1.59 ±.11
L/L (10)	10.0	24.0#	1.40#

Supplemental Table S2. **Periodogram analysis for adult female P. xylostella locomotor rhythms.** Chi-square periodogram analysis of 6-day data sets for the indicated number (n) of individual males under the indicated conditions. Relative rhythmic power (RRP) was calculated as the ratio between chi-square periodogram amplitude and significance threshold. # indicates that there was only a single rhythmic individual.

Condition	M Rhythm *P*	F Rhythm *P*	M Mesor	F Mesor	M Amplitude	F Amplitude	M Phase peak	F Phase peak
D/D	n/a	n/a	n/a	n/a	n/a	n/a	n/a	n/a
12/12	4.05^{-8}	6.61^{-8}	1.67	1.44	1.48	1.24	4.16	5.00
6/6/6/6	3.44^{-13}	9.37^{-15}	1.51	1.20*	1.49	1.11*	5.47	6.23
14/10	6.01^{-6}	3.38^{-8}	1.09	1.05	1.08	1.21	2.73	4.23
16/8	1.31^{-8}	3.58^{-11}	1.50	1.59	1.76	1.68	3.17	3.39
18/6	2.50^{-6}	1.86^{-6}	1.06	1.11	1.15	1.08	2.29	2.06
20/4	1.50^{-4}	2.72^{-5}	1.11	1.05	1.27	1.29	1.52	1.49
L/L	n/a	n/a	n/a	n/a	n/a	n/a	n/a	n/a

Supplemental Table S3. Comparative analysis adult male and female locomotor rhythms by cosine fitting. Circacompare analysis of adult *P. xylostella* male and female locomotor rhythms under various conditions. Significantly different rhythmic features (6/6/6/6 Mesor and Amplitude) are denoted by *. Rhythm *P* equates to the *P* value of data's fit to cosinudsoidal curve, Mesor equates to a rhythm-adjusted mean, Amplitude is a measure of amount of change between peak and trough of calculated curve, Phase peak equates to how many h after dark phase onset is the peak of fitted curve.

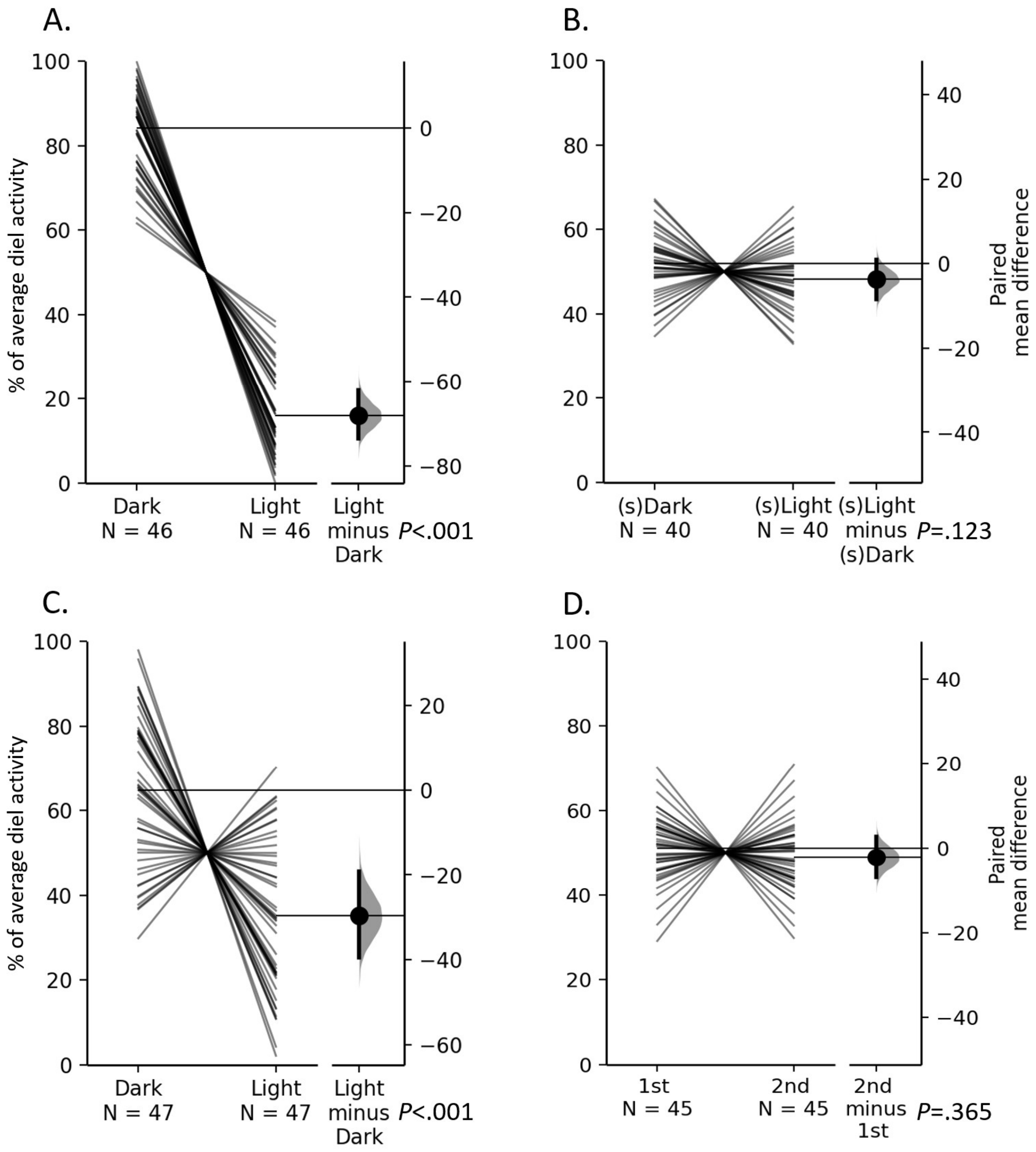

Supplemental Figure S3. Phase-associated locomotor activity differences. Tufte slope graph comparisons are shown for adult *P. xylostella* moth locomotor activity between (A) dark and light phases in 12:12 L/D cycles, (B) dark and subjective dark phases in DD cycles, (C) dark and light phases in 20:4 L/D cycles and (D) the 1st and 2nd 6:6 L/D phases in 6:6:6:6 L/D/L/D cycles. N indicates number of moths with the paired mean difference plotted on the right y-axis with bootstrap sampling distribution and 95% confidence interval indicated by the

vertical error bar with two-sided permutation t-test *P* value.

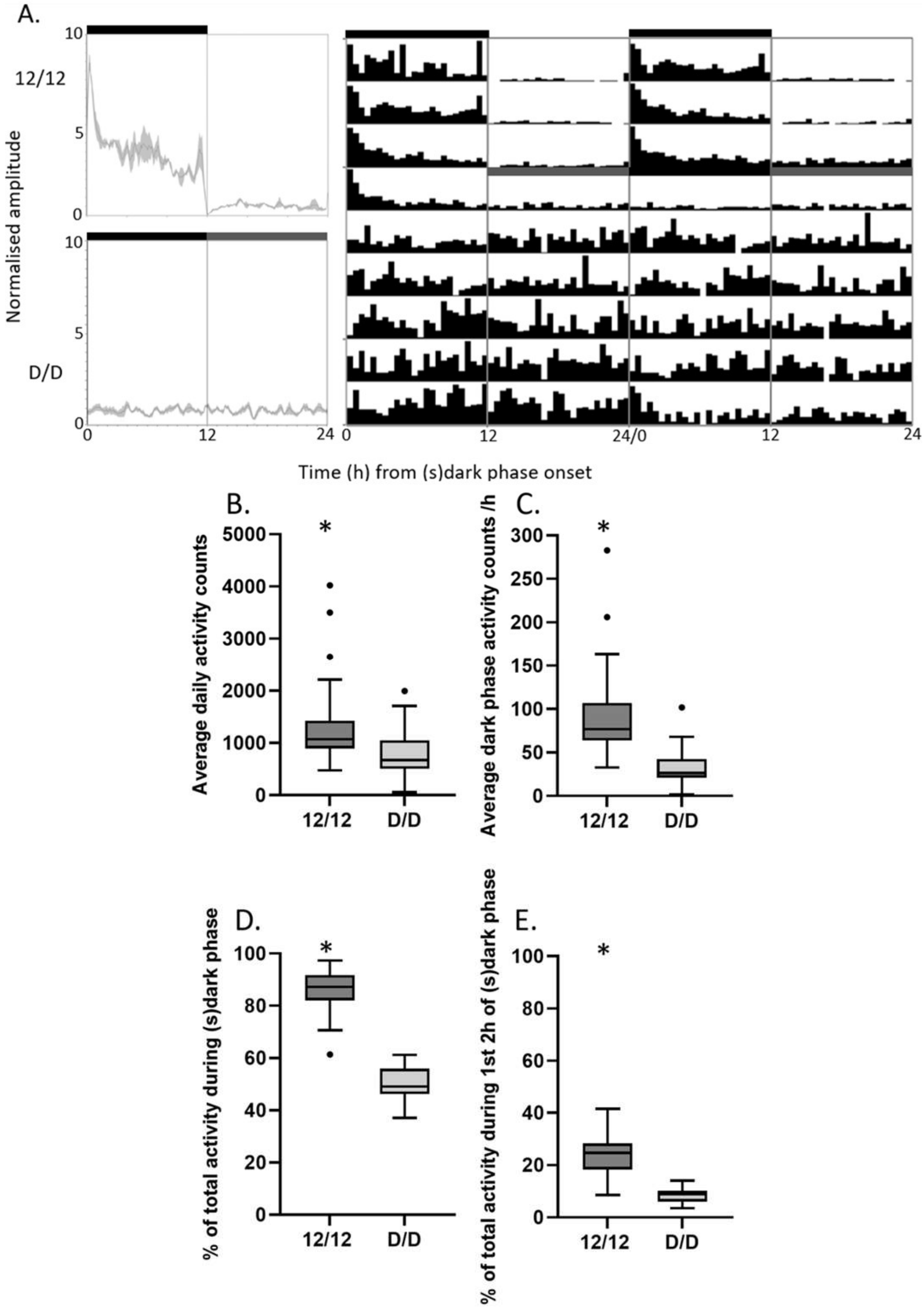

Supplemental Figure S4. Adult male P. xylostella in 20°C under 12/12 and D/D light conditions.

A) Diel activity profiles (Left) show the average activity ± SEM 3-day L/D 12:12 or 6-day D/D intervals. Time is plotted along x-axis in hours (h), starting at dark phase and subjective (s) dark phase onset with normalized amplitude along y-axis. The D/D average starts from first complete day in D/D. The black horizontal bar along the top of individual graphs marks dark phase with white and grey bars representing the L/D light phase and D/D subjective light phase, respectively. Double-plotted actograms (Right) show normalized average activity of moths over in 30 minute bins with the transition to D/D occurring after 3 L/D days. B) Average diel activity, C) average dark phase activity rate per h, D) average % of diel activity occurring during L/D dark phase or D/D subjective dark phase, E) average % of diel activity occurring during first 2h of (s)dark phase. Data points outside Tukey range (1.5XIQR (Interquartile range)) are shown. Mann-Whitney U test results show significant differences from each respective data set through *.

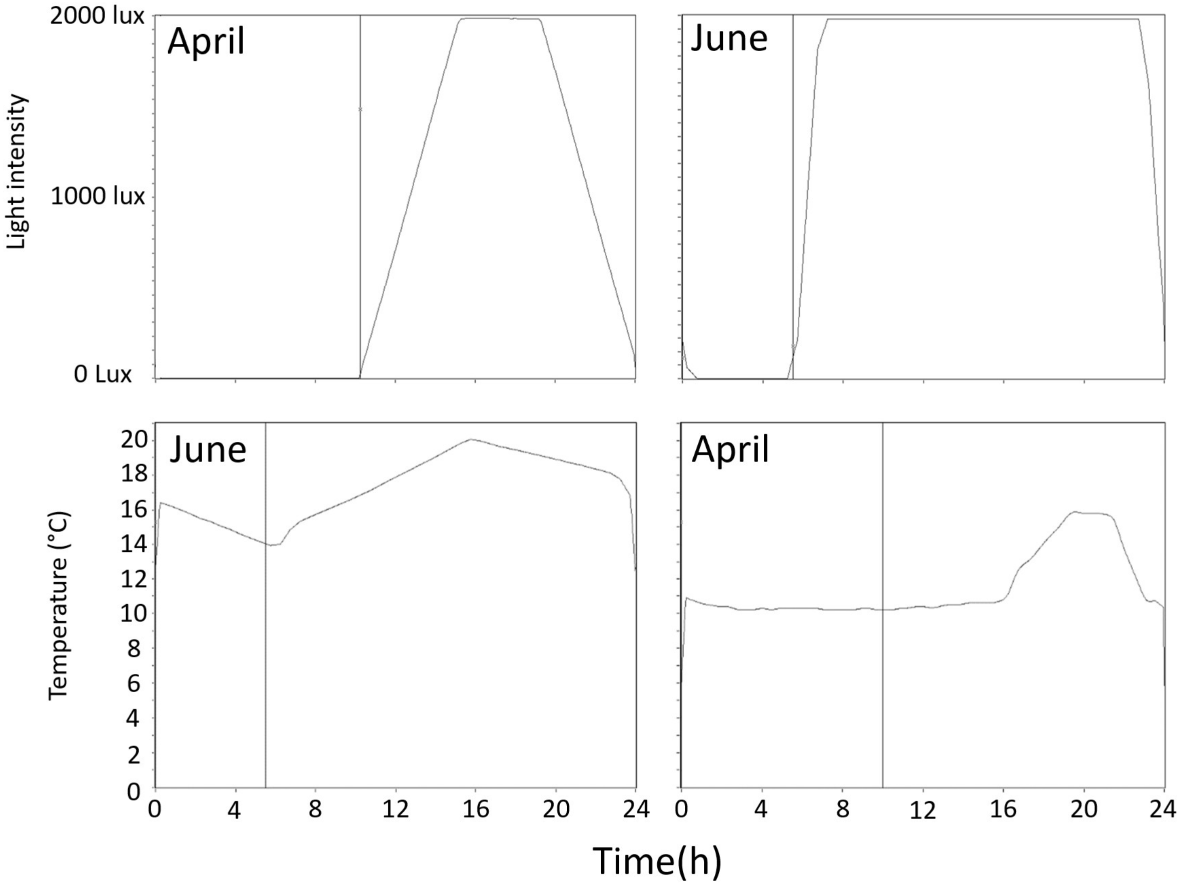

Supplemental Figure S5. **June and April mimic light and temperature profiles**. Environmental profiles were recorded by a DEnM (Drosophila Environment Monitor by TriKinetics) inside Percival incubators set using environmental data from [1]. Drop offs in temperature near beginning and end of temperature profiles is an artifact of analysis software presentation averaging data from environmental monitor across multiple days.

Supplemental References

1. Shaw, B.; Fountain, M.; Wijnen, H. Control of Daily Locomotor Activity Patterns in Drosophila suzukii by the Circadian Clock, Light, Temperature and Social Interactions. *J Biol Rhythms* **2019**, 748730419869085, doi:10.1177/0748730419869085.
